# Effect of Flame Retardant (BDE-47) Exposure on Benthic Organisms from Coastal Areas: Experiment on Symbiont-Bearing Foraminifera of Genus *Peneroplis*

**DOI:** 10.3390/toxics14050441

**Published:** 2026-05-15

**Authors:** Marianna Musco, Marilena Vita Di Natale, Marco Torri, Tiziana Masullo, Carmelo Daniele Bennici, Angela Cuttitta

**Affiliations:** 1 National Research Council—Institute of Studies on the Mediterranean (CNR-ISMed), Palermo’s Office, Via Filippo Parlatore 65, 90145 Palermo, Italy; marianna.musco@cnr.it (M.M.); marilena.dinatale@ismed.cnr.it (M.V.D.N.); tiziana.masullo@cnr.it (T.M.); carmelodaniele.bennici@cnr.it (C.D.B.); angela.cuttitta@cnr.it (A.C.); 2 GEC Department (Law, Economics and Communication), Libera Università Maria Santissima Assunta (LUMSA), Palermo’s Office, Via Filippo Parlatore 65, 90145 Palermo, Italy

**Keywords:** BDE-47, symbiont-bearing foraminifera, sensitivity experiment, vitality indicators

## Abstract

Benthic foraminifera, single-cell marine organisms found worldwide, represent an important component of seabed ecosystems. Due to their sensitivity to environmental pollution, they are often used as bioindicators, providing an efficient tool in toxicity studies. Among the pollutants affecting marine coastal and estuarine environments, persistent flame retardants, such as polybrominated diphenyl ethers (PBDEs), are frequently found. Low-level exposure to BDE-47, a PBDE congener, is known to affect organismal development. In this framework, this study aims to assess the effects of BDE-47 exposure on benthic foraminifera from coastal marine environments. Foraminifera specimens belonging to the symbiont-bearing Peneroplidae family were sampled and exposed to two different BDE-47 concentrations for up to 48 h. Vitality indicators such as changes in pseudopodial activity, movement, reproduction, loss of symbiont algae, and occasional mortality events were monitored during the experiment. Exposure to BDE-47 induced alterations in pseudopodial activity, movement, reproduction, and symbiont retention, with the progressive loss of vitality and limited mortality at increasing exposure levels, highlighting the sensitivity of this species to BDE-47. These findings suggest the harmful repercussions of PBDE pollution on marine coastal ecosystems, affecting benthic organisms and potentially contributing to biomagnification processes within the food web, with possible implications for human health.

## 1. Introduction

Polybrominated diphenyl ethers (PBDEs) are a class of brominated flame retardants extensively incorporated into polymers, textiles, and electronic equipment to reduce flammability. Among them, 2,2′,4,4′-tetrabromodiphenyl ether (BDE-47) is one of the most frequently detected congeners in marine waters and sediments worldwide. This widespread occurrence is mainly due to their continuous and slow release from treated materials, in which PBDEs are not chemically bound to polymer matrices and can gradually leach into the environment during use, weathering, and disposal. Their persistence is further enhanced by their hydrophobic nature and resistance to photolytic, chemical, and biological degradation, which promote long-term stability and bioaccumulation in aquatic and terrestrial environments.

The ecotoxicological concern associated with PBDEs stems from their environmental persistence, potential for long-range transport, and ability to accumulate and biomagnify through aquatic food webs. These characteristics pose significant risks to both ecosystems and human health. Regulatory bodies such as the European Food Safety Authority (EFSA) have highlighted potential reproductive, neurodevelopmental, and endocrine-disruptive effects associated with PBDE exposure in vertebrate models and human exposure pathways, particularly through diet (i.e., seafood consumption) and, to a lesser extent, through water and air [1].

Due to their strong affinity for particulate matter, PBDEs tend to accumulate in marine sediments, making the benthic compartment a major environmental sink for these and other hydrophobic organic contaminants. In this context, benthic foraminifera (forams, for short) are unicellular marine organisms widely distributed in seabed environments and play a key role in ecosystem functioning and trophic networks. They contribute to carbonate production and nutrient cycling and serve as a trophic resource for meio- and macrofaunal consumers, thereby linking sediment processes to higher trophic levels. Thus, in contaminated marine benthic environments, foraminifera are among the first organisms to respond to pollution and can accumulate and transfer contaminants within the marine food web. 

For these reasons, foraminifera are widely used as bioindicators in ecotoxicological studies. Changes in abundance, reduced diversity, shell deformities, and shifts toward stress-tolerant assemblages have been consistently associated with anthropogenic stressors such as heavy metals, organic contaminants, and sediment quality deterioration [2,3,4,5,6]. Within this group, the genus *Peneroplis* Montfort, 1808 [7] comprises medium-to-large symbiont-bearing benthic foraminifera that inhabit shallow photic coastal environments. Their symbiosis with algae enhances metabolic performance but also increases sensitivity to environmental stressors that disrupt host–symbiont interactions, including bleaching-like phenomena observed in other symbiotic marine organisms [8]. Consequently, their use in toxicity studies can provide valuable insights into pollutant impacts on shallow marine ecosystems. 

Despite extensive evidence of PBDE contamination in sediments and marine biota, the mechanistic understanding of their effects on microscopic benthic organisms remains limited. Experimental studies have shown that environmentally relevant PBDE concentrations can affect larval settlement, alter biofilm-associated microbial communities, and disrupt developmental processes in marine invertebrates, suggesting potential sub-lethal impacts across multiple phyla [9,10,11].

Laboratory culture experiments on benthic foraminifera are particularly useful for linking pollutant exposure to specific biological responses under controlled conditions, complementing field-based observations [12]. Although benthic foraminifera have been extensively used as indicators of heavy metal and organic pollution [6,13], their responses to persistent hydrophobic organic pollutants such as PBDEs remain poorly investigated. The present study aims to address this knowledge gap by quantifying physiological and behavioural responses to acute exposure to BDE-47 in *Peneroplis* spp.

Specifically, this study focuses on key functional endpoints, including pseudopodial activity, locomotion, symbiont retention, and asexual reproduction, which reflect different levels of biological organization. Pseudopodial activity provides insight into cytoskeletal dynamics and cellular motility, both highly sensitive to membrane-active contaminants. Locomotor behaviour represents an integrated functional response and is often used as an early indicator of sub-lethal stress. Symbiont retention reflects the stability of the host–symbiont association, which is particularly vulnerable to oxidative imbalance and metabolic disruption. Finally, asexual reproduction represents a population-relevant endpoint closely linked to organismal fitness and energy allocation.

Importantly, this study is designed to investigate early physiological and behavioural responses under environmentally realistic exposure scenarios, rather than to quantify acute lethality endpoints or establish definitive toxicological thresholds. The selected endpoints specifically target sensitive functional traits that can serve as early warning indicators of stress, capturing sub-organismal and organismal processes that may precede more severe toxicological effects. Together, these endpoints provide a mechanistic framework to assess how flame retardant contamination may impact benthic protists and, by extension, broader coastal ecosystem functioning.

## 2. Materials and Methods

### 2.1. Sampling Area and Organism Collection

Ninety *Peneroplis* spp. specimens, belonging to *Peneroplis pertusus* (Forsskål in Niebuhr, 1775) [14] and *Peneroplis planatus* (Fichtel & Moll, 1798) [15], were sampled on 17 May 2021 by manual picking at the lagoon-like coastal basin Stagnone di Marsala (Tp, Italy), in 1 m depth. Sampling was performed on shallow rocky and algal substrates under natural photic conditions. Specimens were transported to the laboratory in in situ seawater within insulated containers to minimize thermal stress. Upon arrival, individuals were gently rinsed with filtered seawater (0.45 μm) and placed in filtered seawater under controlled laboratory conditions, replicating their natural environment (temperature: 20 ± 1 °C; salinity: 39.30 PSU; photoperiod: 15:9 light–dark cycle).

All *Peneroplis* specimens selected for the experiment were adults, a life stage reached once their shells have completed development (starting at about 3 to 5 months of age). Adult shapes are characteristic for each species (ranging from lenticular to spherical in the case of *P. pertusus* or flat and fan-shaped in *P. planatus* [16,17]); adults hosting symbiotic algae have reached sexual maturity [18,19].

*Peneroplis* spp., as well as other foraminifera, have a life cycle alternating between two generations (asexual and sexual reproduction); this cycle lasts about one year, with spring to late summer representing the reproductive season [20].

In this study, the specimens were collected shortly before the reproductive phase of their life cycle.

### 2.2. Experimental Design

After acclimation (24 h), specimens were distributed into nine glass Petri dishes (30 mL each), with 10 individuals per dish. Individuals were visually inspected under a stereomicroscope to confirm vitality (presence of coloured cytoplasm and active pseudopodia). The experimental setup consisted of three groups (N = 3 replicates per group): control (CTL) with filtered seawater without contaminants; low concentration of BDE-47 (0.05 µg/L); high concentration of BDE-47 (0.1 µg/L). To prevent physical interference during the experiments, the three Petri dishes prepared for each treatment were assigned to different observation protocols. The selected concentrations correspond to 5-fold (0.05 µg/L) and 10-fold (0.1 µg/L) increases over the national environmental quality threshold value (based on European Environmental Quality Standards (EQS) for PBDEs in surface waters as sum of priority congeners including BDE-47; 0.014 µg/L) under Directive 2013/39/EU [21].

2,2′,4,4′-tetrabromodiphenyl ether (BDE-47; purity ≥ 97.0%) was purchased from Sigma-Aldrich (St. Louis, MO, USA). A stock solution was prepared at a concentration of 1000 µg/L by dissolving BDE-47 powder in 0.1% DMSO (dimethyl sulfoxide; ≥99.5%, Sigma-Aldrich). The stock solution was stored in amber glass vials at 4 °C until use.

Working solutions (0.05 and 0.1 µg/L) were freshly prepared by diluting the stock solution in filtered seawater. The final DMSO concentration in the exposure media was 0.1% (*v*/*v*), and the same solvent concentration was maintained across treatments to ensure comparability. This concentration was selected as it is widely recognized as a standard maximum threshold for avoiding non-specific solvent-induced artifacts in aquatic ecotoxicity assays [22,23]. An additional solvent control group (filtered seawater + 0.1% DMSO) was also prepared and monitored for physiological and vitality indicators (pseudopodial activity, symbiont stability, and reproduction). Since no differences were observed between the seawater control and the solvent control for these parameters, the seawater control (CTL) was used as the single reference group for all subsequent analyses and graphical representations. Control groups were exposed to filtered seawater without PBDE addition. All solutions were prepared in glassware to minimize the adsorption of the hydrophobic compound onto plastic materials.

All experimental units were maintained under static exposure conditions for 48 h.

Additionally, to assess the presence of BDE-47, a blue-violet filter (400–450 nm) was used, corresponding to the emission peak of the contaminant (410 nm), and observations were conducted at the defined time points described below. Control specimens were included to rule out autofluorescence, which was negligible under these conditions.

Two Petri dishes (representing a pooled population of N = 20 individuals) per treatment and control were observed at five time points: T0 (0 h: 8 pm), T12 (12 h: 8 am), T24 (24 h: 8 pm), T36 (36 h: 8 am) and T48 (48 h: 8 pm). At each time point, vitality indicators and physiological responses, such as changes in pseudopodial activity, loss of symbiont algae and reproduction, were assessed. However, to provide a clear and concise visualization of the overall physiological trends, these parameters are graphically presented at the main daily milestones: initial stage (T0), Day 1 (corresponding to T24), and Day 2 (corresponding to T48).

The third Petri dish for each treatment and control (N = 10 individuals) was strictly dedicated to continuous video recording in order to track foraminiferal movements throughout the experiment (from T0 to T48).

Assessments of vitality indicators, physiological responses, and locomotion are detailed in the following paragraphs.

### 2.3. Evaluation of Pseudopodial Activity

Specimens were checked under an optical microscope in order to determine the presence and extent of pseudopods. Because pseudopodial activity was assessed as a discrete categorical event (presence/absence) rather than a continuous measurement, this parameter did not follow a normal distribution and was therefore recorded and expressed as the frequency of occurrence (percentage) within the population.

### 2.4. Fluorescence Microscopy Analysis of Symbiont Algae and Reproduction Events

Specimens were examined under a fluorescence microscope using a red filter (635–780 nm) to detect chlorophyll autofluorescence from symbiont algae, which was used as a proxy for symbiont presence and holobiont integrity. This signal was also indirectly used to identify reproductive events (schizogony), based on the known transfer of symbionts to daughter cells during reproduction. All microscope and camera settings (exposure and illumination intensity) were kept constant across treatments to allow for comparisons.

Similarly to pseudopodial activity, symbiont presence (chlorophyll autofluorescence) and reproductive occurrence are categorical variables. Due to the discrete nature of these data and the constraints of the sample size, their assessment was not subjected to separate statistical hypothesis testing. Instead, these frequencies were evaluated through an integrated qualitative biological interpretation to provide a comprehensive overview of the organism’s physiological state.

### 2.5. Image-Based Tracking and Statistical Evaluation of Locomotor Behaviour

Locomotor activity was recorded using three action cameras with time-lapse imaging (one frame every 5 min) to reconstruct individual trajectories. Thus, three Petri dishes, one per treatment, each containing ten individuals of *Peneroplis* spp., were continuously monitored for 48 h using the three action cameras.

Locomotor activity was analyzed separately for each of the two daytime monitoring days (Day 1 on 18 May 2021; Day 2 on 19 May 2021) using the following time windows:

- CTL: Day 1 from 09:20 to 20:30; Day 2 from 05:40 to 19:15.
- The 0.05 µg/L group: Day 1 from 05:35 to 20:25; Day 2 from 05:45 to 19:10.
- The 0.1 µg/L group: Day 1 from 05:45 to 20:30; Day 2 from 05:45 to 19:20.

Foraminiferal tracking was performed in Fiji/ImageJ (Version 1.53c) [24] by extracting x–y coordinates for each individual across frames. From individual trajectories, locomotion descriptors were computed separately for Day 1 and Day 2: (i) mean distance travelled between consecutive frames (5 min interval) and (ii) maximum velocity (peak speed) reached within each day.

Unlike the categorical biological parameters (fluorescence and reproduction), locomotor descriptors provided continuous quantitative data suitable for parametric statistical testing. All statistical analyses for locomotion were conducted in R (Version 4.5.0) [25]. Prior to analysis, the assumptions of normality and homoscedasticity were verified using Shapiro–Wilk and Levene’s tests, respectively. As the data satisfied these assumptions (*p* > 0.05 in both tests), the analysis was conducted on raw values. For each locomotion descriptor, differences among groups were tested using a two-way analysis of variance (ANOVA) with treatment (CTL, 0.05 µg/L, 0.1 µg/L) and monitoring day (Day 1, Day 2) as fixed factors, including their interaction (day × treatment). Tukey’s HSD post hoc tests were used for pairwise comparisons. Statistical significance was set at α = 0.05.

## 3. Results

**Figure 1:**
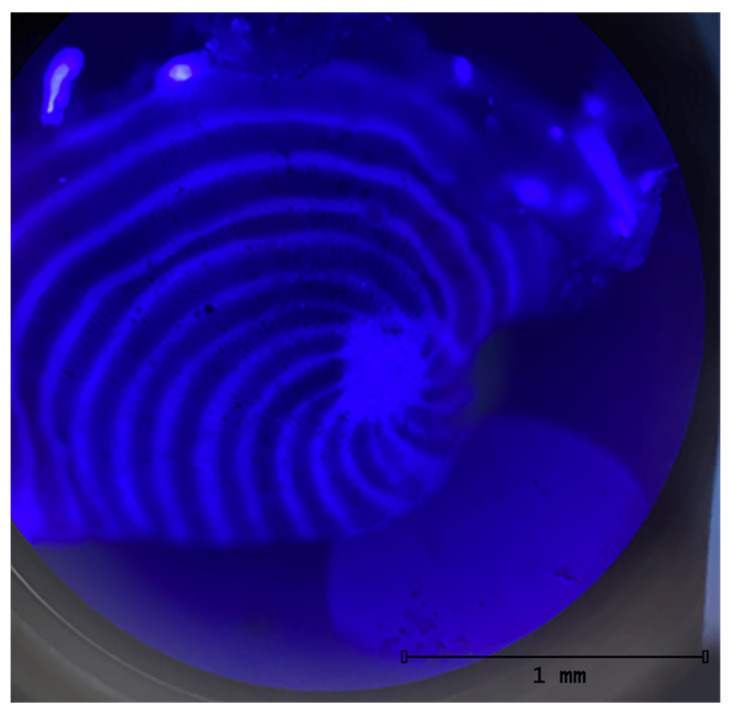
Living specimens of *Peneroplis planatus* from 0.1 ug/L BDE-47 concentration, T12.

To evaluate the effects of BDE-47 exposure on the physiology and biology of symbiont-bearing benthic foraminifera, key vitality indicators were monitored on a total of 90 specimens over a 48 h experimental period. After exposure, the foraminifera were observed under a fluorescence microscope to assess the presence of the contaminant. According to Shan et al. [26], the fluorescence emission wavelength of BDE-47 is 410 nm; therefore, specimens were observed using the blue-violet filter (400–450 nm). All the contaminated specimens showed a vivid blue-violet signal, while the specimens from the control Petri dish did not (Figure 1).

Pseudopodial activity, symbiont retention, reproduction, and locomotor behaviour were analyzed, as these parameters are closely linked to cellular functionality, metabolic balance, and stress response. The following sections describe the observed alterations in these biological traits across the control (CTL) and exposed groups (0.05 µg/L; 0.1 µg/L), with particular emphasis on the potential dose-dependent effects associated with BDE-47 exposure.

### 3.1. Pseudopodial Activity

**Figure 2:**
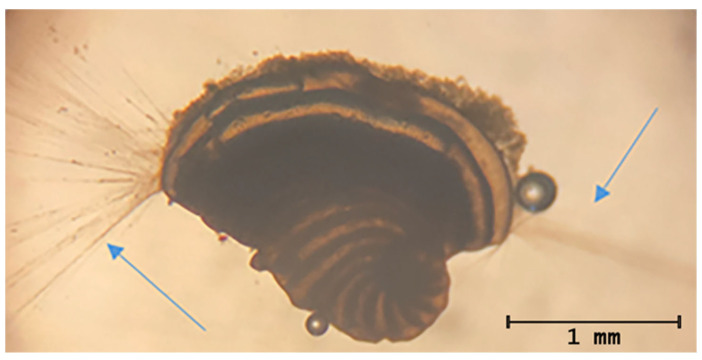
Pseudopodal presence highlighted by the blue arrows.

Pseudopods are everted cellular structures used by foraminifera for substrate attachment, locomotion, food uptake and inter-individual interaction. Their presence reflects organismal vitality and functional integrity (Figure 2).

**Figure 3:**
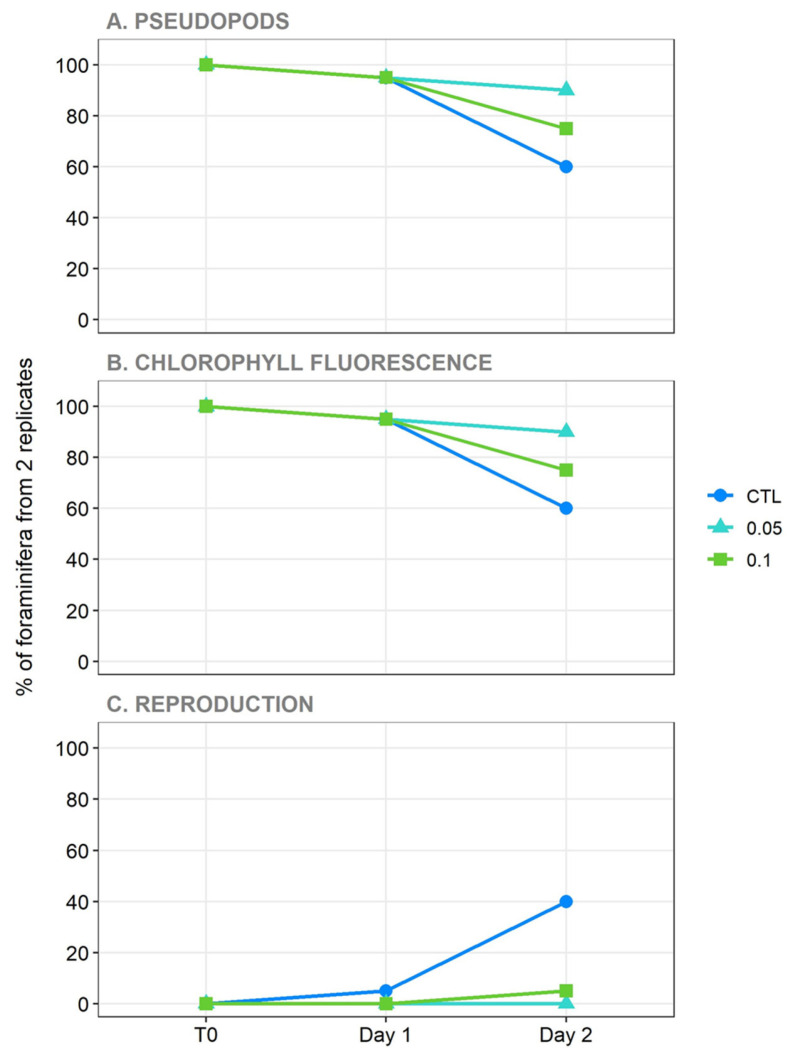
The effects of BDE-47 exposure on 20 individuals of *Peneroplis* spp. per treatment over time. The linecharts illustrate the percentage of individuals (y-axis) showing (**A**) external pseudopods, (**B**) chlorophyll fluorescence, and (**C**) reproduction. Data are reported for the control group (CTL) and two BDE-47 concentrations (0.05 and 0.1 µg/L) at 0, 24, and 48 h (T0, Day 1 and Day 2, respectively).

During the experimental period, the specimens actively utilized their pseudopods to adhere to the Petri dish and interact with their surroundings. As shown in Figure 3A, external pseudopods were consistently present across all experimental groups (CTL, 0.05, and 0.1 µg/L) during the first 24 h (Day 1). This stable activity suggests that exposure to BDE-47 during this time did not trigger acute pseudopodial retraction. However, after 48 h (Day 2), a generalized decline in pseudopodial extension was observed. In the CTL group, this reduction was associated with asexual reproduction, as shown in Figure 3C: pseudopodia were no longer present, and the mother cell had undergone schizogony, as evidenced by daughter cells within and/or around the empty mother shell. In contrast, in the exposed groups, pseudopodial activity declined, but reproduction did not increase accordingly. This decline was more pronounced at the highest BDE-47 concentration (0.1 µg/L), where the number of active individuals decreased compared to both the control and the lower concentration. This delayed response suggests progressive cytoskeletal impairment and severe metabolic stress induced by prolonged exposure to the toxicant.

### 3.2. Symbiont Algae and Reproduction

**Figure 4:**
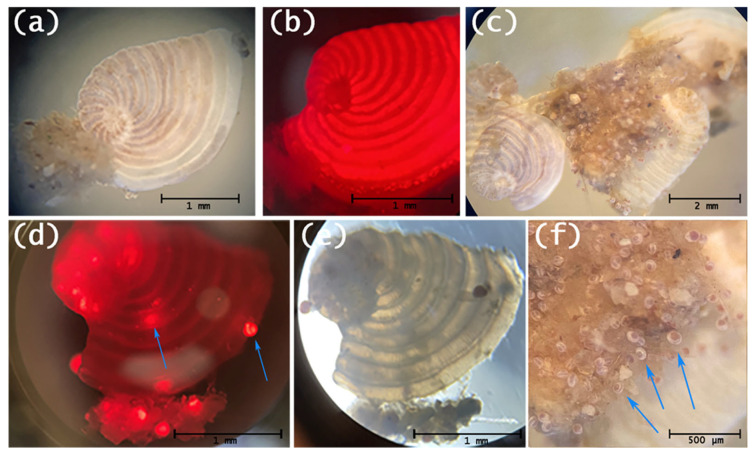
(**a**) Living specimen of *Peneroplis planatus* with symbiont algae within its cell, conferring purplish-violet pigmentation within chambers; optical microscope image, reflected light. (**b**) Chlorophyll red emission by symbiont algae in living foraminifer; fluorescence microscope image. (**c**) Asexual reproduction (schizogony) showing empty mother shells (right) and released daughter cells, alongside non-reproducing individuals with natural violet-hued chambers (left); optical microscope image, reflected light. (**d**) Fluorescence and (**e**) optical microscope image (transmitted light) showing symbiont algal migration within daughter cells (indicated by arrows) inside empty mother shell. (**f**) Detail from image in (**c**): daughter cells with their symbiotic algae (indicated by arrows); optical microscope image, reflected light.

After 12 h on Day 1, asexual reproduction (schizogony) was observed in the control group, as indicated by the presence of daughter cells retaining chlorophyll autofluorescence, confirming the successful symbiont transfer and the physiological integrity of the holobiont. No reproductive events were observed in the exposed groups at this stage. By Day 2, 8 out of 20 control specimens had reproduced asexually (Figure 3C and Figure 4). Conversely, reproductive activity was strongly suppressed in treated groups, with only one reproductive event observed at 0.1 µg/L (Figure 4).

The absence of a chlorophyll autofluorescence signal, interpreted as symbiont loss, was observed in one specimen at both 0.1 and 0.05 µg/L on Day 1, increasing to two specimens at 0.05 µg/L and four at 0.1 µg/L on Day 2 (Figure 3B). This pattern suggests an early destabilization of the host–symbiont relationship under BDE-47 exposure, potentially linked to stress or membrane dysfunction induced by the pollutant.

Overall, these findings indicate a strong inhibitory effect of BDE-47 on reproduction by Day 2. Furthermore, they suggest that symbiont loss in exposed individuals reflects physiological stress rather than the natural reproductive dispersal observed in the control group.

### 3.3. Locomotor Behaviour

**Figure 5:**
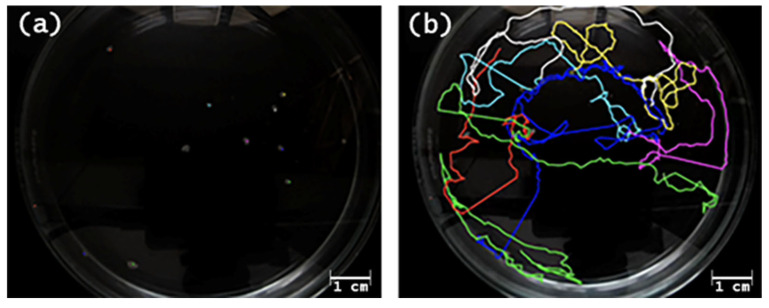
Representative locomotor tracking of ten specimens from control group (filtered seawater). (**a**) Initial positions of individuals at start of experiment (T0); (**b**) cumulative trajectories recorded over 48 h period (T48), where each colour identifies distinct path of individual specimen.

The frame-by-frame tracking of *Peneroplis* spp. specimens resulted in the generation of a locomotion map, as shown in Figure 5.

**Figure 6:**
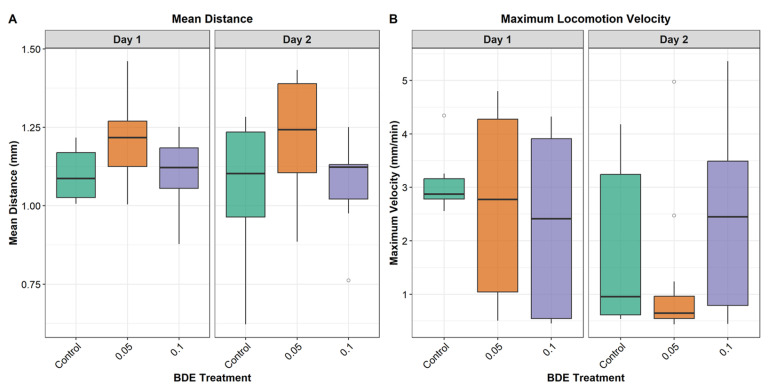
Locomotor responses of *Peneroplis* spp. under BDE-47 exposure during daytime monitoring. Boxplots show (**A**) mean distance travelled between consecutive frames (5 min interval) and (**B**) maximum velocity (peak speed) for individual specimens recorded on Day 1 and Day 2. Treatments include control (CTL) and BDE-47 exposures at 0.05 and 0.1 µg/L.

Locomotion was quantified by calculating the mean distance travelled between consecutive frames (5 min intervals) and the maximum velocity reached by each individual. Both parameters were extracted separately for the first and second days of monitoring, considering only daytime recording hours. These measurements produced the locomotion responses presented in Figure 6.

Statistical analyses were performed to assess the presence of significant differences among experimental groups. For these analyses, the individual was treated as the statistical replicate (N = 10 per treatment). Although individuals were housed in the same Petri dish per treatment, the risk of pseudoreplication was considered negligible due to the large volume of the arena (30 mL) relative to the organisms’ size and the absence of social or schooling behaviours in *Peneroplis* spp. In particular, two-way analyses of variance (ANOVA) were conducted to test the effects of experimental day and treatment condition (control vs. BDE exposures), as well as their interaction (day × treatment), on locomotion parameters. This approach allowed us to evaluate whether behavioural responses varied between monitoring days and among exposure levels and whether treatment effects differed depending on the day of observation.

The two-way ANOVA showed that the mean distance travelled differed significantly among BDE treatments (F = 6.07, *p* = 0.004), while neither the effect of monitoring day (F = 0.14, *p* = 0.706) nor the treatment × day interaction (F = 0.22, *p* = 0.804) was significant. Tukey post hoc tests indicated that individuals in the 0.05 µg/L exposure travelled a greater mean distance than controls (mean difference = 0.141 mm, 95% CI = 0.031–0.251, *p* = 0.0087). Mean distance did not differ between 0.1 µg/L and the control (*p* = 0.980), whereas the 0.1 µg/L group travelled significantly less than the 0.05 µg/L group (mean difference = −0.132 mm, 95% CI = −0.242 to −0.022, *p* = 0.0149).

For maximum velocity, treatment had no significant effect (F = 0.80, *p* = 0.457), whereas monitoring day was significant (F = 4.62, *p* = 0.036); peak velocities were lower on Day 2 than on Day 1 (mean difference = −0.799 mm min^−1^, 95% CI = −1.544 to −0.053, *p* = 0.036). The treatment × day interaction was not significant (F = 2.02, *p* = 0.143), and Tukey post hoc comparisons did not identify significant pairwise differences among treatments for maximum velocity (all adjusted *p* > 0.46).

## 4. Discussion

The present study demonstrates that short-term exposure to sub-lethal concentrations of BDE-47 induces measurable physiological and behavioural alterations in symbiont-bearing benthic foraminifera of the genus *Peneroplis*. The observed response, including reduced pseudopodial activity, inhibition of asexual reproduction, altered locomotor patterns, and symbiont loss, collectively indicates a complex, concentration-specific stress response consistent with early sub-lethal toxicity. The tested concentrations (0.05 and 0.1 µg/L) are within the range of regulatory concern for PBDEs in aquatic systems and can be related to environmental quality standards established for surface waters [21]. Although environmental concentrations are often lower, higher levels have been reported in contaminated coastal sediments and biota, supporting the ecological relevance of the selected exposure range. Compared to previous experimental studies on marine organisms, which frequently employ higher concentrations, the present work specifically targets sub-lethal thresholds.

Here, sub-lethal concentrations refer to exposure levels not associated with immediate or mass mortality but inducing measurable physiological and behavioural responses. Although the tested concentrations were selected to represent sub-lethal exposure conditions, limited mortality was observed at the highest concentration and at later exposure times. It is important to clarify that the experimental design was not intended to derive quantitative lethality thresholds (i.e., LC50) but rather to assess early physiological and behavioural responses to contaminant exposure. The occurrence of mortality under these conditions suggests that prolonged exposure, even at concentrations considered sub-lethal, may ultimately lead to lethal outcomes, reinforcing the relevance of the observed sub-lethal endpoints as early warning signals of toxic effects.

### 4.1. Pseudopodial Impairment and Cytophysiological Stress

Foraminiferal exposure to BDE-47 resulted in a marked reduction in pseudopodal activity, indicating a clear impairment of a fundamental function underlying motility, feeding, and environmental interaction. This response suggests that even at sub-lethal levels, BDE-47 can significantly affect cellular performance and organismal functionality. Such impairment is likely linked to the disruption of processes regulating cytoskeletal dynamics (actin polymerization) and intracellular signalling [27,28], although the present data do not allow for the identification of a specific primary target. Given the lipophilic nature of PBDEs and their known capacity to interact with cellular membranes, membrane-associated effects represent a plausible upstream mechanism, potentially affecting downstream calcium-dependent signalling pathways and cytoskeletal organization. Comparable sub-lethal endpoints have been described in benthic foraminifera exposed to other contaminants, such as heavy metals and hydrocarbons, where decreased pseudopodial extension has been interpreted as an early indicator of metabolic distress and cytological disruption [13,29]. Our findings are consistent with this interpretation and extend it to PBDE exposure. Specifically, a comparison between the present results on BDE-47 exposure and previous studies on cadmium (Cd) toxicity in benthic foraminifera of the species *Ammonia* cf. *parkinsoniana* [30] reveals both convergent responses and key differences in toxicological pathways. In both cases, a reduction in pseudopodial activity emerges as a sensitive and early indicator of stress. In Cd-exposed *specimens*, this response is dose- and time-dependent; in contrast, the impairment observed in BDE-47-exposed *Peneroplis* spp. occurs under sub-lethal conditions and is interpreted as a functional disruption affecting motility, feeding, and environmental interaction. This difference reflects distinct modes of action: Cd primarily induces direct cytotoxic effects on cellular metabolism, whereas BDE-47 likely acts through membrane-mediated processes, altering intracellular signalling and cytoskeletal dynamics.

Accordingly, whereas Cd toxicity is well framed within established environmental thresholds, the effects observed here for BDE-47 highlight the ecological relevance of sub-lethal endpoints, emphasizing the need to integrate functional responses into ecotoxicological assessments.

In a broader context, the reduction in pseudopodial activity observed in *Peneroplis* spp. likely reflects an early functional decline preceding more severe physiological effects. This is supported by the co-occurrence of additional stress-related responses in the present study, including altered locomotor behaviour, reduced reproductive output, and symbiont destabilization, suggesting a systemic impact on organismal fitness.

### 4.2. Reproductive Inhibition and Population-Level Implications

A marked reduction in asexual reproduction (schizogony) was recorded at the highest BDE-47 concentration, with the complete and near-complete suppression of cell division in the 0.05 and 0.1 µg/L treatments, respectively. This response indicates a strong sensitivity of reproductive processes to contaminant exposure, suggesting that energy allocation and/or cell cycle progression are among the earliest biological functions affected. Comparable inhibitory effects on reproduction have been reported in other marine organisms exposed to BDE-47 [31], including rotifers and copepods, where reduced fecundity and altered population growth parameters were observed. For instance, in the rotifer *Brachionus plicatilis*, exposure to similar levels resulted in reduced fecundity and altered population growth parameters, indicating that reproductive processes are particularly sensitive to PBDE contamination even at low concentrations [32,33]. At higher exposure levels, consistent effects have also been observed in the copepod *Nitocra spinipes*, where PBDE exposure led to reduced development, reproduction, and population growth rates, supporting the view that these compounds can impair key life-history traits across a wide concentration range. In the present work, under control conditions, successful reproduction and symbiont transfer to offspring confirmed the maintenance of physiological and symbiotic integrity. In contrast, the disruption observed under exposure conditions points to a shift in organismal priorities, potentially reflecting a stress-induced reallocation of energy away from reproduction towards maintenance and survival processes. Such responses are consistent with previous findings showing that PBDE exposure can impair reproductive output while affecting physiological balance and oxidative status [34].

Although the exact mechanisms cannot be resolved here, interference with cell cycle regulation and metabolic energy balance represents a plausible explanation, consistent with the systemic effects of lipophilic contaminants on marine protists. In particular, experimental evidence from marine microalgae indicates that BDE-47 can arrest the cell cycle (e.g., at the G2/M phase), directly inhibiting cell division [35].

From an ecological perspective, reproductive output is a critical determinant of population maintenance in benthic foraminifera [36]. Therefore, even modest reductions in division rates may translate into altered community structure over ecological timescales. Reductions in population growth and reproductive output have been reported in copepods exposed to PBDEs, suggesting that these effects can scale up to influence population dynamics [32]. Given the ecological role of larger symbiont-bearing taxa such as *Peneroplis* spp. in carbonate sediment production and benthic trophic transfer, such reproductive impairment may ultimately affect sediment stability and ecosystem functioning [37].

### 4.3. Symbiont Loss as a Stress Biomarker

The symbiont loss observed in exposed specimens represents one of the most ecologically relevant responses to BDE-47 exposure, as it directly reflects the disruption of the tightly integrated host–symbiont system. In *Peneroplis* spp., this association underpins key physiological processes, including photosynthetic carbon fixation, supporting calcification and energy reserves. This mutualistic equilibrium is highly sensitive to oxidative imbalance [8], and the magnitude of symbiont reduction observed in the present study is consistent with the early-stage disruption of this balance rather than complete bleaching, as described in ultrastructural investigations [5,38,39].

In this study, symbiont presence was assessed indirectly through chlorophyll autofluorescence, and its reduction or loss therefore reflects decreased symbiont retention within the host. Studies on foraminifera exposed to environmental stressors such as elevated temperature have demonstrated that symbiont loss is a common outcome of physiological disruption, with responses varying according to exposure intensity, duration, and organismal tolerance [40]. While such studies typically describe organism-level bleaching responses under environmental forcing, the response observed here follows a clear dose-dependent pattern across contaminant concentrations, indicating a progressive impairment of the symbiotic association at the functional level. Although the underlying mechanisms were not directly observed, oxidative imbalance represents a plausible driver of this response. In this context, the present work captures an earlier functional signal of symbiotic disruption, where BDE-47 exposure induces a measurable impairment of symbiont-associated chlorophyll fluorescence at sub-lethal concentrations, extending previous evidence on contaminant-driven oxidative stress.

BDE-47 exposure is known to induce oxidative stress across a range of marine organisms, typically assessed through biochemical or transcriptomic endpoints [41,42]. Our results provide a functional, organism-level manifestation of this stress, linking oxidative imbalance to symbiont loss. Similar pathways have been described in foraminifera exposed to trace metals, where oxidative stress correlates with physiological impairment [43], although these studies generally rely on enzymatic biomarkers rather than symbiosis-based endpoints. The consistency between these approaches suggests that the reduction in chlorophyll fluorescence observed here may represent an integrated response to oxidative disruption. Oxidative stress can destabilize symbiosome membranes, impair host–symbiont communication, and promote algal expulsion [44,45]. While such cellular processes were not directly observed in this study, the progressive decrease in symbiont-associated fluorescence is consistent with these mechanisms. Functionally, symbiont depletion reduces autotrophic energy input, potentially accelerating physiological decline under prolonged exposure [45,46]. This phenomenon is consistent with bleaching-like responses described in other marine symbiotic systems subjected to chemical or thermal stress, reinforcing the ecological relevance of this endpoint.

Beyond foraminifera, evidence from other marine taxa further supports oxidative imbalance as a conserved response to BDE-47 exposure. In this context, the reduction in chlorophyll autofluorescence observed in *Peneroplis* spp. is consistent with physiological responses associated with oxidative stress documented in other marine organisms exposed to BDE-47 [47,48,49]. For instance, in sea urchin embryos, BDE-47 exposure has been shown to affect the transcriptional modulation of genes involved in oxidative balance, stress response, and developmental regulation, although the tested concentrations were one to two orders of magnitude higher than those used here. These findings support the hypothesis that oxidative imbalance represents a key mode of action of this congener and suggest that the symbiont depletion observed in *Peneroplis* spp. may reflect a shared stress response pathway across different taxa.

### 4.4. Locomotor Responses as Early Behavioural Endpoints

Locomotor analyses provide a functional readout consistent with the progressive stress phenotype inferred from pseudopodial activity, symbiont stability, and reproductive output. The mean distance travelled between consecutive 5 min frames differed among treatments, with individuals exposed to 0.05 µg/L showing significantly greater displacement than controls, whereas the 0.1 µg/L group travelled significantly less than the 0.05 µg/L group and did not differ from controls. This pattern indicates a non-linear behavioural response across exposure levels. This non-linear response is consistent with biphasic dose response patterns frequently described in ecotoxicology, where low-dose stimulation followed by inhibition at higher exposure levels reflects a hormesis-like mechanism of physiological compensation and subsequent constraint [50]. Similar patterns have been reported in aquatic organisms exposed to organic contaminants, where behavioural activation at low concentrations is interpreted as an initial compensatory response before energetic or cellular limitation [51,52].

For the mean distance travelled, neither the effect of monitoring day nor the day × treatment interaction was significant, indicating that treatment-related differences were comparable across observation periods. In contrast to sustained displacement, maximum velocity was not significantly affected by treatment but declined from Day 1 to Day 2, indicating reduced burst capacity over time. This decrease is biologically plausible in the context of reduced pseudopodial extension at later time points and may reflect energetic limitation and/or the progressive impairment of cytoskeletal and adhesion efficiency. According to the boxplots (Figure 6), exposure-related shifts in response distributions are observed, including occasional extreme values at the highest concentration. Such behavioural variability has been reported in meiofaunal and protist-based systems exposed to contaminants and may reflect episodic stress-driven movements or reduced regulatory control under stronger physiological challenge than the loss of behavioural control [52]. An experiment conducted on *Platymonas subcordiformis* has highlighted inhibitory effects on movement following exposure to high concentrations of BDE-47 (9.695 mg/L) [53]. Although the concentrations tested on *P. subcordiformis* were higher than those tested on *Peneroplis* spp., both results highlight an impairment of locomotion due to the presence of BDE-47. Overall, these results support locomotion-based metrics as sensitive, non-invasive behavioural endpoints for detecting early sub-lethal contaminant effects in micro- and meiofaunal organisms, often preceding overt morphological alterations.

### 4.5. Ecological and Trophic Implications

The ecological significance of these findings extends beyond individual-level toxicity. PBDEs are persistent, sediment-associated contaminants prone to bioaccumulation and biomagnification along benthic and pelagic food webs. As primary consumers and prey for meiofaunal and macrofaunal organisms, benthic foraminifera may contribute to contaminant transfer across trophic levels.

The sub-lethal impairment of benthic protists could therefore produce dual effects: (i) structural alterations in benthic community composition and carbonate cycling and (ii) the facilitation of contaminant flux toward higher trophic levels. Considering the documented occurrence of PBDEs in coastal sediments and biota, chronic exposure scenarios may pose long-term risks to ecosystem integrity and, indirectly, to human health through seafood consumption pathways. The reported environmental concentrations of BDE-47 are generally low or below detection limits in open marine systems, whereas higher levels have been measured in contaminated coastal areas such as industrialized zones, harbours, and regions affected by wastewater discharge or historical PBDE inputs. The concentrations used in the present study, therefore, reflect worst-case exposure conditions relevant to such localized hotspots rather than background marine environments.

**Figure 7:**
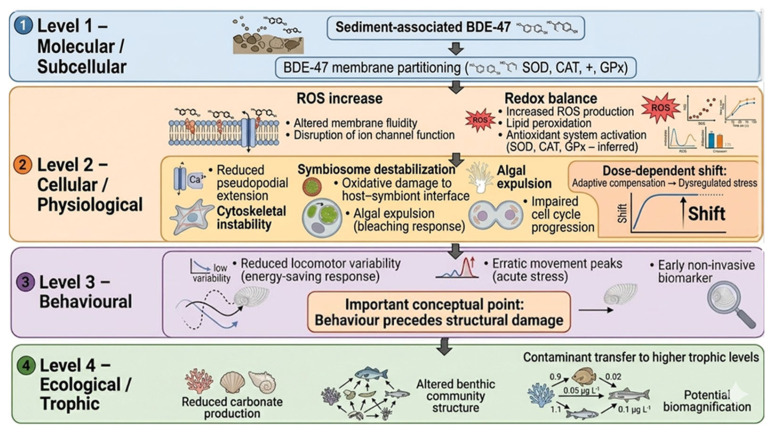
Proposed mechanistic cascade of BDE-47 toxicity in *Peneroplis* spp. across multiple levels of biological organization, from molecular (Level 1) to ecological (Level 4), highlighting potential sub-lethal effects and their propagation across scales. The figure was generated and refined using Google Gemini 3 Flash (Google LLC, Mountain View, CA, USA), specifically utilizing the Nano Banana 2 (Gemini 3 Flash Image) model for image synthesis and text rendering.

Collectively, these findings further underscore the need for future research on spatially explicit exposure gradients to better understand ecological risks under realistic exposure scenarios. Additionally, the present observations support a mechanistic interpretation in which the membrane partitioning of BDE-47 initiates redox imbalance and intracellular signalling disruption at the molecular and subcellular levels (Level 1), ultimately leading to cytoskeletal destabilization and symbiont loss at the cellular and physiological levels (Level 2). These alterations are reflected in behavioural changes, such as a modified locomotor pattern (Level 3), and may contribute to reproductive impairment. Beyond individual-level effects, these functional disruptions may scale up to influence carbonate production, benthic community dynamics, and contaminant transfer across trophic networks at the ecological level (Level 4). A conceptual framework summarizing this cascade of events, from toxic perturbation to organism-level and ecological responses, is presented in Figure 7.

### 4.6. Concluding Remarks

Although this experiment was limited to acute (48 h) exposure, the detected responses highlight the sensitivity of symbiont-bearing foraminifera to environmentally relevant BDE-47 concentrations. Future research should address:

- Chronic and multigenerational exposure effects;
- Quantification of oxidative stress biomarkers (e.g., antioxidant enzyme activity);
- Calcification rate measurements and shell ultrastructural analysis;
- Molecular endpoints (gene expression related to stress and cytoskeletal regulation).

Integrating laboratory bioassays with field-based community analyses would strengthen ecological risk assessment frameworks.

In summary, exposure to BDE-47 induced coordinated morphological, behavioural, and reproductive alterations in *Peneroplis* spp., supporting their suitability as sensitive bioindicators for persistent organic pollutant assessment in coastal marine ecosystems.

## 5. Conclusions

This study represents the first investigation into the effects of BDE-47 contamination on foraminifera.

Sub-lethal exposure to BDE-47 induces measurable morphological and behavioural alterations in symbiont-bearing benthic foraminifera of the genus *Peneroplis*. Observed effects, including reduced pseudopodial activity, reproductive inhibition, and symbiont loss, act as early stress biomarkers and indicate a predominantly dose-dependent pattern of sub-lethal effects in *Peneroplis* spp., although certain endpoints, such as locomotor behaviour, showed a non-monotonic response, suggesting a more complex relationship between exposure level and behavioural response. Such non-monotonic responses are commonly observed in ecotoxicological studies and may reflect the activation of compensatory stress-adaptive mechanisms at intermediate exposure levels.

Given the ecological role of benthic foraminifera in carbonate production and trophic transfer, these findings highlight the potential ecosystem-level consequences of PBDE contamination in coastal environments. Furthermore, considering the biomagnification potential of PBDEs, indirect implications for higher trophic levels and human health cannot be excluded.

This study supports the suitability of symbiont-bearing foraminifera as sensitive bioindicators for assessing the ecotoxicological impact of persistent organic pollutants in shallow marine ecosystems.

## Data Availability

Data are unavailable due to privacy or ethical restrictions.
